# Exploring multilevel social determinants of depressive symptoms for Tanzanian adolescents: evidence from a cross-sectional study

**DOI:** 10.1136/jech-2020-216200

**Published:** 2021-03-29

**Authors:** Leah Prencipe, Tanja AJ Houweling, Frank J van Lenthe, Tia M Palermo, Lusajo Kajula, Adolescent Cash Plus Evaluation Team, Tia Palermo

**Affiliations:** 1 Department of Public Health, Erasmus MC, University Medical Centre, Rotterdam, The Netherlands; 2 Department of Epidemiology and Environmental Health, University at Buffalo, Buffalo, New York, USA; 3 UNICEF Office of Research - Innocenti, Florence, Italy

**Keywords:** depression, adolescents CG, social epidemiology, gender, socio-economic

## Abstract

**Background:**

Depression has substantial and enduring impacts for adolescents, particularly those living in poverty. Yet, evidence on its determinants in low-income countries remains scarce. We examined the social determinants of depressive symptoms for Tanzanian adolescents.

**Methods:**

We used cross-sectional data for 2458 adolescents (aged 14–19), to describe associations with depressive symptoms within and across five domains—demographic, economic, neighbourhood, environmental and social-cultural—using linear mixed models. We estimated depressive symptoms using the 10-item Centre for Epidemiological Studies Depression Scale, which ranges from 0 to 30 and increases with additional symptoms.

**Results:**

Factors associated with depressive symptoms in the fully adjusted models included experiencing five or more household economic shocks (β=2.40; 95% CI 1.48 to 3.32), experiencing droughts/floods (β=0.76; 95% CI 0.36 to 1.17), being in a relationship (β=1.82; 95% CI 1.30 to 2.33), and having moderate (β=1.26; 95% CI 0.80 to 1.71) or low (β=2.27; 95% CI 1.81 to 2.74) social support. Exclusive schooling was protective compared with being engaged in both school and paid work (β=1.07; 95% CI 0.05 to 2.61) and not engaged in either (β=0.73; 95% CI 0.24 to 1.22). Household size and relationship status were more important factors for girls, while employment status, and extreme precipitation were more important for boys.

**Conclusion:**

Mental health is associated with determinants from multiple domains. Results suggest that environmental shocks related to climate change contribute to poor mental health in adolescents, highlighting an important area for intervention and research.

## Introduction

Poor mental health causes the highest disease burden for youth.^1^ Depression in adolescence contributes to diminished educational achievement, substance use, delinquency and suicide, one of the leading causes of mortality for adolescents.^2^ Adolescent-onset mental health disorders often persist through adulthood,^3^ are associated with other poor outcomes later in life,^4^ and perpetuate into future generations.^5^ Poor populations are acutely vulnerable, as social and economic conditions of poverty increase the likelihood of having depressive disorders, and depressive orders themselves decrease overall economic well-being.^6^

Mental health is influenced by numerous interwoven biological and social mechanisms that exist in various domains.^7^ While identifying the social determinants of poor mental health is crucial to inform interventions which target these mechanisms, research gaps on the influence of environmental events, macroeconomic determinants, and household economics remain.^8^ Recent studies have explored determinants of poor mental health among African youth,^9^ although the evidence has largely been focused on specific subgroups, such as youth living with HIV,^10^ school-going children,^11–13^ pregnant women,^14^ and orphans or adults.^15–17^ With the majority of the population younger than 18 years,^18^ understanding the determinants of adolescent mental health in Tanzania is crucial.

Using the conceptual framework developed by Lund *et al*,^7^ we categorise potential determinants of mental health into five domains (demographic, economic, neighbourhood, environmental, and social and cultural). Lund’s conceptual framework follows Bronfenbrenner’s ecological approach to childhood,^19^ wherein proximal, intermediate, and distal factors are conceptualised to capture the complex multidimensional ways in which social determinants influence child health. Despite increased understanding that factors within cultures, families, communities, and environments work together to influence outcomes, ecological approaches to child health are still largely theoretical in studies of African populations.^20^ The evidence remains fixed on attributes and behaviours of individuals and largely ignores the environmental and contextual factors which help shape those individualistic characteristics. Few studies have evaluated such a comprehensive set of social determinants on adolescent mental health in Africa.

## Methods

### Study population

The cross-sectional data come from the evaluation of an adolescent-focused intervention designed to complement the Productive Social Safety Net (PSSN), a governmental social protection programme. Information on the study design and intervention are provided in the evaluation report.^21^ Data were collected in 130 villages from four districts within Iringa and Mbeya regions of mainland Tanzania. All adolescents who (1) were living in a PSSN household and (2) were 14–19 years of age, were eligible for inclusion, resulting in a sample of 2458 youth. While data used in this study come from baseline (April to June 2017), select indicators were only available at the first follow-up (May to June 2018) and included 2104 adolescents.

### Measures

Data come from village, household, and youth surveys, which were translated to Swahili and pilot tested. Informed consent was obtained from respondents 18 or older and married youth; caregiver/parental consent and youth assent were obtained for unmarried youth under 18 years.

#### Mental health

The 10-item Centre for Epidemiological Studies Depression Scale (CES-D10) is an internationally validated screening tool.^22^ Youth responses to 10 questions regarding feelings and behaviours over the prior 7 days (online supplemental table 1) were summed to create a scale ranging from 0 to 30, with higher scores reflecting more depressive symptoms. The CES-D10 has been validated as a reliable measurement of depression in Tanzania, with full measurement invariance of the scale supported by gender.^23^ We further included a binary indicator of ≥10 on the CES-D10 to denote depressive symptomology for descriptive purposes. While a recent validation of the CES-D10 in South Africa recommended cut-offs between 11 and 13, depending on the language of population, the study was unable to estimate the prevalence of depression among adolescents.^24^ Therefore, we used the recommended cut-off, which is the most commonly used threshold among similar populations.^23^

10.1136/jech-2020-216200.supp1 Supplementary data

#### Potential determinants

Indicators were selected based on data availability and overlap with determinants from Lund *et al* 7 online supplemental appendix. Domain definitions from the Lund study and variable construction are provided in online supplemental table 2. The demographic domain included sex, age, female-headed household, household with five or more members, and region. The economic domain included household wealth level (created with an asset-based index), number of household economic shocks experienced during the prior year, and cell phone ownership. The neighbourhood domain included access to basic services level (created with a summary index of resources available within the village). The environmental domain included household affected by drought or flood and household affected by livestock or crop disease, both during the prior year. Finally, the social and cultural domain included education/employment status, relationship status, religious attendance, number of adverse child experiences (ACE) (based on a subset of the ACE International Questionnaire^25^), and level of social support (based on a modified version of the Multidimensional Scale of Perceived Social Support).^26^

#### Psychosocial indicators

Although psychosocial factors are important pathways between determinants and health, we explored these indicators descriptively as they may overlap with other predictors.^27^ Subjective quality of life (QOL), self-esteem", and locus of control (LOC), the extent of feeling internal control over outcomes (eg, a person has control over one’s own life) versus external (ie, life is controlled by outside factors),^28^ have all been strongly associated with mental health.^29 30^ We created levels of self-esteem based on two items from Rosenberg’s self-esteem scale,^31^ poor QOL using a 10-point ‘Cantril’s Ladder of Life Scale’,^32^ and level of LOC based on five items from Levenson’s multidimensional LOC scale,^33^ wherein a higher level indicates more internal control. Details on variable construction of psychosocial indicators can be found in online supplemental table 3.

### Analysis

First, we summarised the potential determinants and psychosocial indicators and reported the unadjusted percent depressed by characteristic, including χ^2^ p values to detect differences in distributions among depressed adolescents. Second, we calculated bivariate associations between each indicator and depressive symptoms using linear mixed models to account for clustering of CES-D10 within and between villages and adjusting for sample stratification indicators (region and village size). Finally, we calculated multivariate associations within and across domains using four linear mixed models. While all multivariate models adjusted for demographics and sampling stratum, model 1 examined economic indicators; model 2 neighbourhood and environmental indicators; model 3 social and cultural indicators; and model 4 combined all domains. The reported intraclass correlation coefficient (ICC) indicates the proportion of the variance on the village level.^34^ As males and females have been shown to process stressors differently, a particularly important factor considering the hormonal fluctuations experienced during adolescence,^35^ results were reported for the full sample and separately by sex. Religious attendance and ACE were not included in multivariate models due to smaller sample size and lack of influential results.

## Results

Of the 2458 youth interviewed at baseline, just over half (1322) were boys and 18% reported being in a relationship (table 1). During the year prior to the survey, nearly one-third (32%) were affected by droughts or flooding and 8% lived in households with five or more economic shocks.

**Table 1:** Characteristics of sample population by domain for the full sample and separately by sex

Domain	Variable	Pooled sample			Females			Males		
		n (% in each category)	% Depressed	χ^2^ P value	n (% in each category)	% Depressed	χ^2^ P value	n (% in each category)	% Depressed	χ^2^ P value
Demographic	Total	2458 (100)	29		1136 (100)	27		1322 (100)	30	
	Sex			0.088						
	Female	1136 (46)	27		–	–		–	–	
	Male	1322 (54)	30		–	–		–	–	
	Age in years			<0.001			0.002			0.142
	14	502 (20)	22		270 (24)	19		232 (18)	25	
	15	498 (20)	26		250 (22)	26		248 (19)	27	
	16	456 (19)	30		200 (18)	29		256 (19)	31	
	17	469 (19)	33		190 (17)	32		279 (21)	33	
	18	296 (12)	30		119 (10)	29		177 (13)	31	
	19	237 (10)	38		107 (9)	38		130 (10)	37	
	Five or more household members			0.013			0.073			0.075
	No	1121 (46)	26		505 (44)	25		616 (47)	28	
	Yes	1337 (54)	31		631 (56)	29		706 (53)	32	
	Female-headed household			0.550			0.548			0.182
	No	840 (34)	30		387 (34)	26		453 (34)	33	
	Yes	1618 (66)	28		749 (66)	28		869 (66)	29	
	Region			<0.001			0.306			<0.001
	Mbeya	1171 (48)	23		538 (47)	29		633 (48)	18	
	Iringa	1287 (52)	34		598 (53)	26		689 (52)	42	
Economic	Wealth level of household			0.387			0.198			0.381
	Richest	818 (33)	29		383 (34)	25		435 (33)	32	
	Middle	819 (33)	31		397 (35)	30		422 (32)	31	
	Poorest	819 (33)	27		355 (31)	27		464 (35)	28	
	Number of economic shocks (past year)			<0.001			<0.001			0.010
	0	626 (25)	27		291 (26)	27		335 (25)	27	
	1	666 (27)	29		309 (27)	27		357 (27)	31	
	2	498 (20)	26		226 (20)	21		272 (21)	30	
	3	299 (12)	24		134 (12)	24		165 (12)	25	
	4	163 (7)	29		73 (6)	15		90 (7)	40	
	5+	206 (8)	48		103 (9)	53		103 (8)	42	
	Youth owns a cell phone			0.028			0.001			0.915
	No	1946 (79)	28		938 (83)	25		1008 (76)	30	
	Yes	512 (21)	33		198 (17)	36		314 (24)	31	
Neighbourhood/ environmental	Access to services level			0.400			0.500			0.513
	High	884 (36)	29		412 (36)	26		472 (36)	31	
	Middle	802 (33)	30		374 (33)	29		428 (32)	31	
	Low	772 (31)	27		350 (31)	26		422 (32)	28	
	Drought/flood (past year)			<0.001			0.296			<0.001
	No	1662 (68)	26		784 (69)	26		878 (66)	26	
	Yes	796 (32)	35		352 (31)	29		444 (34)	40	
	Livestock/crop disease (past year)			0.366			0.607			0.484
	No	1423 (58)	28		672 (59)	27		751 (57)	30	
	Yes	1035 (42)	30		464 (41)	28		571 (43)	31	
Social and cultural	Education/employment			<0.001			<0.001			0.037
	Attending school/training	1254 (51)	24		662 (58)	21		592 (45)	26	
	Engaged in paid work	300 (12)	35%		64 (6)	36		236 (18)	35	
	In school/training and paid work	85 (3)	32		26 (2)	27		59 (4)	34	
	Not in education, employment, or training	819 (33)	34		384 (34)	36		435 (33)	33	
	Has a spouse, boyfriend or girlfriend			<0.001			<0.001			0.002
	No	2023 (82)	26		834 (73)	22		1189 (90)	29	
	Yes	435 (18)	42		302 (27)	42		133 (10)	42	
	Social support			<0.001			<0.001			0.001
	High	951 (39)	22		353 (31)	16		598 (45)	25	
	Moderate	790 (32)	30		368 (32)	27		422 (32)	33	
	Low	717 (29)	37		415 (37)	36		302 (23)	37	
	Attends weekly religious ceremony*			0.338			0.278			0.963
	No	992 (47)	30		374 (39)	29		618 (54)	30	
	Yes	1113 (53)	28		582 (61)	26		531 (46)	30	
	Number of adverse childhood experiences*			0.830			0.296			0.981
	0–1	399 (20)	27		161 (19)	22		238 (22)	31	
	2	549 (28)	30		246 (29)	28		303 (28)	31	
	3	402 (21)	28		172 (20)	26		230 (21)	29	
	4	284 (15)	30		135 (16)	30		149 (14)	30	
	5+	314 (16)	31		144 (17)	33		170 (16)	29	

Depression is defined using a cut-off of 10 or higher on the 10-item Centre for Epidemiological Studies Depression Scale.

*Total N differs due to attrition. Data come from wave 2 for these indicators.

While 29% of the sample reported depressive symptomology, the rates varied by characteristic. Higher rates were found for youth affected by droughts or floods (35%) than not (26%), and for youth with low social support (37%) compared with high (22%). Nearly half of youth with five or more economic shocks reported depressive symptomology (48%). Findings were mostly similar by sex, with some notable distinctions: boys in Iringa had higher rates of depression (42%) when compared with those in Mbeya (18%), depressive symptomology was higher among boys who experienced droughts or floods (40%) than boys who did not (26%), and, among girls, cell phone owners had higher depression (36%), compared with non-owners (25%).

Among psychosocial indicators (table 2), youth with low self-esteem exhibited higher rates of depression (33%) than their counterparts (29% moderate and 25% high), while youth with a high internal LOC had lower rates (19%) than those with moderate (32%) or low (38%) levels. While rates by self-perceived QOL were less different overall, the differences were larger for girls: 32% who reported poor QOL had depressive symptoms compared with 20% for girls who did not. For boys with a low internal LOC, nearly half (46%) reported depressive symptoms, compared with 31% and 18% for those with moderate and high levels, respectively.

**Table 2:** Psychosocial characteristics of sample population for the full sample and separately by sex

Variable	Pooled sample			Females			Males		
	n (% in each category)	% Depressed	χ^2^ P value	n (% in each category)	% Depressed	χ^2^ P value	n (% in each category)	% Depressed	χ^2^ P value
Poor self-perceived quality of life			0.018			<0.001			0.716
No	1130 (46)	27		451 (40)	20		679 (51)	31	
Yes	1328 (54)	31		685 (60)	32		643 (49)	30	
Self-esteem			0.001			<0.001			0.009
High	775 (32)	25		321 (28)	22		454 (34)	27	
Moderate	906 (37)	29		454 (40)	22		452 (34)	36	
Low	777 (32)	33		361 (32)	38		416 (31)	29	
Locus of control			<0.001			0.001			<0.001
High	948 (39)	19		420 (37)	21		528 (40)	18	
Moderate	730 (30)	32		358 (32)	33		372 (28)	31	
Low	780 (32)	38		358 (32)	28		422 (32)	46	

Depression is defined using a cut-off of 10 or higher on the 10-item Centre for Epidemiological Studies Depression Scale.

As seen in the pooled sample, all psychosocial indicators were associated with depressive symptoms (table 3). Having a poor self-perceived QOL was associated with nearly one-point higher CES-D10 than the reference category (β=0.81; 95% CI 0.40 to 1.21), and having a low level of internal LOC was associated with a nearly two-point higher LOC than those with a high level (β=1.98; 95% CI 1.52 to 2.44). Lacking internal LOC and self-esteem were more strongly associated with mental health for boys, while poor QOL was more influential for girls.

**Table 3:** Bivariate associations of psychosocial characteristics and depressive symptoms for the full sample and separately by sex

Variable	Full sample			Females			Males		
	Mean CES-D10	Estimate (95% CI)	P value	Mean CES-D10	Estimate (95% CI)	P value	Mean CES-D10	Estimate (95% CI)	P value
Poor self-perceived quality of life									
No	6.51	Reference category		5.86	Reference category		6.94	Reference category	
Yes	6.86	0.81 (0.40 to 1.21)	<0.001	7.11	1.39 (0.80 to 1.99)	<0.001	6.59	0.29 (−0.25 to 0.84)	0.292
Self-esteem									
High	6.21	Reference category		6.30	Reference category		6.15	Reference category	
Moderate	6.53	0.65 (0.18 to 1.13)	0.007	5.74	−0.30 (−1.01 to 0.41)	0.405	7.33	1.73 (1.12 to 2.34)	<0.001
Low	7.38	1.43 (0.95 to 1.92)	<0.001	7.99	1.82 (1.09 to 2.56)	<0.001	6.84	1.20 (0.58 to 1.82)	<0.001
Locus of control									
High	5.57	Reference category		5.91	Reference category		5.31	Reference category	
Moderate	7.06	1.52 (1.06 to 1.98)	<0.001	7.23	1.31 (0.63 to 2.00)	<0.001	6.90	1.33 (0.72 to 1.93)	<0.001
Low	7.73	1.98 (1.52 to 2.44)	<0.001	6.82	0.91 (0.22 to 1.61)	0.010	8.50	2.24 (1.62 to 2.86)	<0.001

Reported CES-D10 means are unadjusted. Bivariate regressions tested associations of psychosocial characteristics with depressive symptoms and adjusted for stratification variables. Linear mixed models were used to account for clustering of CES-D10 within and between villages. Random effects are not shown.

CES-D10, 10-item Centre for Epidemiological Studies Depression Scale.

### Social determinants of depressive symptoms

#### Demographic domain

Increased age was associated with higher CES-D10 in bivariate analyses for the full sample (table 4). This association was partly (and for girls fully) explained by social and cultural characteristics (model 3, online supplemental tables 4 and 5). While living in a household with five or more people had no clear association with CES-D10 in the full sample, girls from large households had higher CES-D10 in all models (online supplemental table 5), including model 4 (β=0.62; 95% CI 0.06 to 1.18). Living in Iringa also had a modest association with depressive symptoms in bivariate regressions (table 4; β=0.56; 95% CI −0.03 to 0.75), with results driven by boys (table 5; β=1.68; 95% CI 0.79 to 2.57). Boys overall had higher CES-D10 than girls in Model 4 (table 4; β=0.70; 95% CI 0.31 to 1.10) but not in bivariate regressions.

**Table 4:** Bivariate and multivariate associations of potential determinants and depressive symptoms, full sample

Domain	Variable	Mean CES-D10	Bivariate		Multivariate model 4	
			Estimate (95% CI)	P value	Estimate (95% CI)	P value
Demographic	Total	6.70				
	Sex					
	Female	6.77	Reference category		Reference category	
	Male	6.61	0.20 (−0.19 to 0.58)	0.314	0.70 (0.31 to 1.10)	<0.001
	Age in years					
	14	5.75	Reference category		Reference category	
	15	6.39	0.62 (0.02 to 1.22)	0.043	0.60 (0.03 to 1.18)	0.039
	16	6.69	0.96 (0.35 to 1.57)	0.002	0.73 (0.14 to 1.32)	0.016
	17	7.17	1.39 (0.79 to 2.00)	<0.001	0.95 (0.33 to 1.58)	0.003
	18	7.25	1.53 (0.84 to 2.22)	<0.001	0.86 (0.10 to 1.62)	0.027
	19	7.76	1.87 (1.13 to 2.62)	<0.001	1.09 (0.26 to 1.92)	0.010
	Five or more household members					
	No	6.46	Reference category		Reference category	
	Yes	6.90	0.36 (−0.03 to 0.75)	0.073	0.37 (−0.01 to 0.76)	0.057
	Female-headed household					
	No	6.71	Reference category		Reference category	
	Yes	6.69	0.04 (−0.37 to 0.45)	0.847	0.16 (−0.24 to 0.56)	0.426
	Region*					
	Mbeya	6.31	Reference category		–	
	Iringa	7.06	0.56 (−0.05 to 1.17)	0.074	–	
Economic	Wealth level of household					
	Richest	6.54	Reference category		Reference category	
	Middle	6.77	0.40 (−0.10 to 0.90)	0.114	0.45 (−0.02 to 0.93)	0.063
	Poorest	6.78	0.41 (−0.13 to 0.95)	0.137	0.47 (−0.05 to 0.99)	0.076
	Number of economic shocks (past year)					
	0	6.57	Reference category		Reference category	
	1	6.63	0.03 (−0.50 to 0.56)	0.916	0.01 (−0.51 to 0.54)	0.964
	2	6.22	−0.21 (−0.79 to 0.36)	0.466	−0.39 (−1.03 to 0.24)	0.223
	3	6.23	−0.19 (−0.87 to 0.48)	0.573	−0.08 (−0.87 to 0.70)	0.834
	4	6.76	0.07 (−0.76 to 0.91)	0.860	0.35 (−0.58 to 1.28)	0.463
	5+	9.10	2.72 (1.95 to 3.49)	<0.001	2.40 (1.48 to 3.32)	<0.001
	Youth owns a cell phone					
	No	6.57	Reference category		Reference category	
	Yes	7.20	0.68 (0.20 to 1.15)	0.005	−0.44 (−1.01 to 0.12)	0.123
Neighbourhood/environmental	Access to services level					
	High	6.69	Reference category		Reference category	
	Middle	6.83	−0.05 (−0.78 to 0.69)	0.899	−0.03 (−0.72 to 0.65)	0.927
	Low	6.57	0.12 (−0.62 to 0.87)	0.747	0.02 (−0.68 to 0.72)	0.947
	Drought/flood (past year)					
	No	6.35	Reference category		Reference category	
	Yes	7.42	0.93 (0.51 to 1.34)	<0.001	0.76 (0.36 to 1.17)	<0.001
	Livestock/crop disease (past year)					
	No	6.59	Reference category		Reference category	
	Yes	6.84	0.36 (−0.04 to 0.75)	0.075	−0.25 (−0.78 to 0.28)	0.354
Social and cultural	Education/employment					
	Attending school/training	6.07	Reference category		Reference category	
	Engaged in paid work	7.22	1.38 (0.76 to 1.99)	<0.001	0.56 (−0.10 to 1.23)	0.098
	School & paid work	7.20	1.28 (0.23 to 2.34)	0.017	1.07 (0.05 to 2.09)	0.040
	Neither	7.42	1.47 (1.04 to 1.90)	<0.001	0.73 (0.24 to 1.22)	0.003
	Has a partner					
	No	6.35	Reference category		Reference category	
	Yes	8.34	2.01 (1.52 to 2.51)	<0.001	1.82 (1.30 to 2.33)	<0.001
	Social support					
	High	5.69	Reference category		Reference category	
	Moderate	6.76	1.37 (0.91 to 1.83)	<0.001	1.26 (0.80 to 1.71)	<0.001
	Low	7.97	2.41 (1.94 to 2.88)	<0.001	2.27 (1.81 to 2.74)	<0.001
	Attends weekly religious ceremony†					
	No	6.73	Reference category		–	
	Yes	6.65	−0.23 (−0.64 to 0.19)	0.285	–	
	Number of adverse childhood experiences†					
	0–1	6.77	Reference category		–	
	2	6.52	−0.24 (−0.86 to 0.39)	0.454	–	
	3	6.55	−0.23 (−0.90 to 0.44)	0.503	–	
	4	6.76	−0.05 (−0.79 to 0.70)	0.903	–	
	5+	6.93	0.13 (−0.60 to 0.85)	0.727	–	
Random effects						
Village variance (SE)			–		1.41 (0.325)	
Intraclass correlation coefficient %			–		6.5	

Reported CES-D10 means are unadjusted. All regressions adjust for stratification variables. Multivariate model 4 adjusts for all domains within a single regression. Models 1–3, which adjust for domains separately, are shown in the appendix. Linear mixed models were used to account for clustering of CES-D10 within and between villages (random effects are not shown for bivariate regressions).

*All other bivariate and multivariate regressions adjust for region as part of sample stratification; therefore estimates are not shown elsewhere.

†Total N differs due to attrition. Data come from wave 2 for these indicators.

CES-D10, 10-item Centre for Epidemiological Studies Depression Scale.

**Table 5:** Bivariate and multivariate associations of potential determinants and depressive symptoms, by sex

Domain	Variable	Females					Males				
		Mean	Bivariate		Multivariate model 4		Mean	Bivariate		Multivariate model 4	
		CES-D10	Estimate (95% CI)	P value	Estimate (95% CI)	P value	CES-D10	Estimate (95% CI)	P value	Estimate (95% CI)	P value
Demographic	Age in years										
	14	5.77	Reference category		Reference category		5.73	Reference category		Reference category	
	15	6.12	0.16 (−0.67 to 1.00)	0.699	0.34 (−0.45 to 1.13)	0.401	6.66	0.92 (0.10 to 1.74)	0.028	0.85 (0.05 to 1.64)	0.037
	16	6.58	0.63 (−0.26 to 1.52)	0.163	0.57 (−0.27 to 1.41)	0.184	6.77	1.08 (0.27 to 1.89)	0.009	0.93 (0.14 to 1.73)	0.022
	17	7.09	1.16 (0.26 to 2.06)	0.011	0.50 (−0.41 to 1.41)	0.278	7.22	1.29 (0.50 to 2.09)	0.001	1.21 (0.38 to 2.04)	0.004
	18	7.95	2.04 (1.00 to 3.09)	<0.001	0.98 (−0.15 to 2.11)	0.089	6.77	1.07 (0.18 to 1.96)	0.018	0.75 (−0.24 to 1.74)	0.138
	19	7.63	1.82 (0.72 to 2.91)	0.001	0.39 (−0.85 to 1.62)	0.539	7.88	1.94 (0.95 to 2.92)	<0.001	1.72 (0.62 to 2.81)	0.002
	Five or more household members										
	No	6.21	Reference category		Reference category		6.67	Reference category		Reference category	
	Yes	6.94	0.63 (0.05 to 1.21)	0.033	0.62 (0.06 to 1.18)	0.029	6.87	−0.10 (−0.60 to 0.41)	0.702	−0.10 (−0.60 to 0.41)	0.703
	Female-headed household										
	No	6.39	Reference category		Reference category		6.99	Reference category		Reference category	
	Yes	6.73	0.26 (−0.35 to 0.87)	0.401	0.30 (−0.28 to 0.89)	0.305	6.66	−0.18 (−0.71 to 0.35)	0.514	−0.02 (−0.55 to 0.51)	0.933
	Region*										
	Mbeya	6.90	Reference category		–		5.80	Reference category		–	
	Iringa	6.35	−0.60 (−1.32 to 0.13)	0.106	–		7.67	1.68 (0.79 to 2.57)	<0.001	–	
Economic	Wealth level of household										
	Richest	6.36	Reference category		Reference category		6.70	Reference category		Reference category	
	Middle	6.79	0.54 (−0.17 to 1.26)	0.137	0.44 (−0.23 to 1.11)	0.193	6.76	0.24 (−0.43 to 0.91)	0.477	0.39 (−0.26 to 1.04)	0.239
	Poorest	6.69	0.26 (−0.52 to 1.03)	0.517	0.27 (−0.46 to 1.01)	0.464	6.84	0.37 (−0.35 to 1.08)	0.311	0.42 (−0.29 to 1.12)	0.245
	Number of economic shocks (past year)										
	0	6.65	Reference category		Reference category		6.50	Reference category		Reference category	
	1	6.54	−0.16 (−0.93 to 0.62)	0.693	−0.36 (−1.13 to 0.40)	0.353	6.71	0.17 (−0.53 to 0.86)	0.643	0.45 (−0.25 to 1.15)	0.209
	2	5.69	−1.07 (−1.92 to −0.23)	0.013	−1.39 (-2.30 to −0.47)	0.003	6.67	0.40 (−0.34 to 1.15)	0.288	0.57 (−0.27 to 1.41)	0.181
	3	6.49	−0.32 (−1.32 to 0.68)	0.528	−0.46 (−1.61 to 0.69)	0.430	6.01	−0.02 (−0.90 to 0.86)	0.958	0.59 (−0.46 to 1.63)	0.271
	4	5.49	−1.45 (-2.70 to −0.21)	0.022	−1.24 (-2.62 to 0.14)	0.079	7.79	1.37 (0.30 to 2.44)	0.012	1.89 (0.69 to 3.09)	0.002
	5+	9.70	2.70 (1.57 to 3.82)	<0.001	1.96 (0.63 to 3.29)	0.004	8.50	2.10 (1.07 to 3.14)	<0.001	2.43 (1.19 to 3.67)	<0.001
	Youth owns a cell phone										
	No	6.35	Reference category		Reference category		6.77	Reference category		Reference category	
	Yes	7.88	1.54 (0.79 to 2.29)	<0.001	−0.44 (−1.01 to 0.12)	0.123	6.77	0.15 (−0.44 to 0.75)	0.612	−0.61 (−1.32 to 0.10)	0.094
Neighbourhood/ environmental	Access to services level										
	High	6.41	Reference category		Reference category		6.93	Reference category		Reference category	
	Middle	6.96	0.45 (−0.41 to 1.31)	0.303	0.24 (−0.54 to 1.03)	0.543	6.72	−0.56 (−1.64 to 0.51)	0.305	−0.32 (−1.34 to 0.70)	0.533
	Low	6.48	0.06 (−0.83 to 0.94)	0.902	−0.18 (−1.00 to 0.63)	0.659	6.65	0.18 (−0.92 to 1.27)	0.751	0.22 (−0.82 to 1.26)	0.683
	Drought/flood (past year)										
	No	6.40	Reference category		Reference category		6.31	Reference category		Reference category	
	Yes	7.09	0.57 (−0.06 to 1.20)	0.075	0.42 (−0.19 to 1.02)	0.180	7.68	1.15 (0.62 to 1.69)	<0.001	1.03 (0.51 to 1.56)	<0.001
	Livestock/crop disease (past year)										
	No	6.48	Reference category		Reference category		6.70	Reference category		Reference category	
	Yes	6.81	0.11 (−0.48 to 0.71)	0.714	−0.12 (−0.89 to 0.66)	0.762	6.87	0.38 (−0.12 to 0.89)	0.139	−0.60 (−1.30 to 0.09)	0.088
Social and cultural	Education/employment										
	Attending school/training	5.94	Reference category		Reference category		6.21	Reference category		Reference category	
	Engaged in paid work	7.44	1.60 (0.35 to 2.84)	0.012	0.26 (−1.00 to 1.53)	0.683	7.17	1.21 (0.50 to 1.93)	0.001	0.63 (−0.15 to 1.40)	0.113
	School & paid work	6.15	0.29 (−1.60 to 2.19)	0.761	0.24 (−1.55 to 2.04)	0.791	7.66	1.70 (0.47 to 2.93)	0.007	1.41 (0.22 to 2.61)	0.021
	Neither	7.67	1.78 (1.17 to 2.40)	<0.001	0.51 (−0.23 to 1.25)	0.178	7.20	1.08 (0.50 to 1.66)	<0.001	0.66 (0.02 to 1.31)	0.043
	Has a partner										
	No	5.91	Reference category		Reference category		6.65	Reference category		Reference category	
	Yes	8.56	2.79 (2.15 to 3.43)	<0.001	2.23 (1.58 to 2.88)	<0.001	7.84	1.30 (0.48 to 2.13)	0.002	1.07 (0.23 to 1.90)	0.012
	Social support										
	High	5.34	Reference category		Reference category		5.90	Reference category		Reference category	
	Moderate	6.32	1.17 (0.45 to 1.89)	0.002	0.86 (0.17 to 1.56)	0.015	7.14	1.42 (0.83 to 2.00)	<0.001	1.31 (0.74 to 1.88)	<0.001
	Low	7.96	2.53 (1.83 to 3.23)	<0.001	2.20 (1.52 to 2.88)	<0.001	7.98	2.22 (1.59 to 2.86)	<0.001	2.11 (1.47 to 2.74)	<0.001
	Attends weekly religious ceremony†										
	No	6.90	Reference category		–		6.63	Reference category		–	
	Yes	6.40	−0.51 (−1.14 to 0.12)	0.116	–		6.92	0.17 (−0.37 to 0.70)	0.542	–	
	Number of adverse childhood experiences†										
	0–1	6.35	Reference category		–		7.05	Reference category		–	
	2	6.49	0.26 (−0.71 to 1.23)	0.596	–		6.53	−0.40 (−1.17 to 0.38)	0.318	–	
	3	6.42	0.10 (−0.95 to 1.15)	0.850	–		6.66	−0.04 (−0.88 to 0.80)	0.925	–	
	4	6.58	0.28 (−0.84 to 1.40)	0.625	–		6.93	0.18 (−0.78 to 1.13)	0.716	–	
	5+	7.28	1.17 (0.05 to 2.28)	0.040	–		6.64	0.01 (−0.90 to 0.92)	0.982	–	
Random effects											
Village variance (SE)				–	1.02 (0.438)				–	3.63 (0.744)	
Intraclass correlation coefficient %				–	4.9				–	16.8	

Reported CES-D10 means are unadjusted. All regressions adjust for stratification variables. Multivariate model 4 adjusts for all domains within a single regression. Models 1–3, which adjust for domains separately, are shown in the appendix. Linear mixed models were used to account for clustering of CES-D10 within and between villages (random effects are not shown for bivariate regressions).

*All other bivariate and multivariate regressions adjust for Region as part of sample stratification; therefore estimates are not shown elsewhere.

†Total N differs due to attrition. Data come from wave 2 for these indicators.

CES-D10, 10-item Centre for Epidemiological Studies Depression Scale.

#### Economic domain

Five or more economic shocks were associated with nearly three points higher CES-D10 in bivariate regressions (table 4; β=2.72; 95% CI 1.95 to 3.49), when compared with no shocks. Controlling for other determinants did not mitigate this relationship, as seen in model 4 (β=2.40; 95% CI 1.48 to 3.32) and when disaggregating by sex (table 5). However, results on fewer than five shocks varied for girls—in bivariate regressions girls who experienced four shocks had a 1.45 points lower CES-D10 (95% CI –2.70 to –0.21), than those that had no shocks. These results were consistent across multivariate models (online supplemental table 5). Household wealth was associated with CES-D10 when adjusting for demographics only (model 1, online supplemental table 4), with no clear associations when adjusting for all domains. Owning a cell phone was associated with more symptoms for girls in bivariate regressions (table 5), however, this relationship was explained by demographics (model 1, online supplemental table 5).

#### Neighbourhood and environmental domains

Youth living in households affected by droughts or floods had nearly one-point higher CES-D10 than those who did not in bivariate regressions (table 4; β=0.93; 95% CI 0.51 to 1.34). Adjusting for demographics (online supplemental table 4, model 1; β=0.91; 95% CI 0.49 to 1.33) or all domains (model 4; β=0.76; 95% CI 0.36 to 1.17) did not affect this relationship. The association between extreme precipitation and depressive symptoms was driven by boys (online supplemental table 6), as we can see a one-point increase in CES-D10 after adjusting for demographics (model 2; β=1.15; 95% CI 0.61 to 1.69) and in the fully adjusted model for boys (β=1.03; 95% CI 0.51 to 1.90), and no relationship for girls (table 5).

#### Social and cultural domain

Having a romantic partner was strongly associated with depressive symptoms overall in bivariate regressions (table 4; β=1.47; 95% CI 1.04 to 1.90). Controlling for other domains only increased the strength of this relationship: youth with romantic partners had nearly a two-point higher CES-D10 (β=1.82; 95% CI 1.30 to 2.33) when compared with single youth in model 4. While results were consistent by sex, the association was stronger for girls (table 5). Not having a high social support level was associated with depressive symptoms overall, with model 4 (table 4) showing increased CES-D10 for youth with moderate (β=1.26; 95% CI 0.80 to 1.71) and low (β=2.27; 95% CI 1.81 to 2.74) levels. While exclusive school had a protective association in bivariate regressions, when compared with engaged in paid work (β=1.38; 95% CI 0.76 to 1.99); both school and paid work (β=1.28; 95% CI 0.23 to 2.34); and neither (β=1.47; 95% CI 1.04 to 1.90), the relationship was mitigated when including other domains, resulting in a negligible association for youth in paid work and reduced associations for those in both school and paid work (β=1.07; 95% CI 0.05 to 2.09) and neither (β=0.73; 95% CI 0.24 to 1.22), in the final model. Associations with employment status were stronger for boys (table 5). Experiencing five or more adverse childhood experiences was associated with more depressive symptoms for girls only (β=1.17; 95% CI 0.05 to 2.28).

#### Village variance

The ICC ranges from 6.5% to 7.4% in full sample multivariate models, as seen in the bottom rows of online supplemental table 4. Results by sex indicate that the village-level variance accounted for a much larger proportion of depressive symptoms for boys, with an ICC three times that of girls in the final model (table 5) (ICC 16.8% vs 4.9%, respectively).

## Discussion

This paper has examined social determinants of adolescent mental health for a vastly understudied and high-risk group. Consistent with existing evidence on the multifactorial causes of poor mental health, we found that depressive symptoms were associated with social determinants across domains. Depressive symptoms were associated with demographic (increased age, being male), economic (five or more economic shocks), environmental (droughts/floods), and social and cultural (romantic partnerships, education/employment status, social support) determinants, in the fully adjusted model. Although all psychosocial indicators were associated with depressive symptoms, the association was stronger among boys lacking internal LOC and self-esteem, while poor QOL was more influential for girls. Neighbourhood access to services had no association, although multilevel models suggest village variance contributed to individual-level depressive symptoms.

The proportion of variance attributable to the village-level remained stable, accounting for 6.9% of symptoms in the empty model (not shown) and 6.5% in the final model, meaning that our selected characteristics do not explain away the village-level contribution to depressive symptoms. When conducting observational research on neighbourhood-level health effects, it may be preferable to select outcome-specific resources as opposed to proxy indicators with wide-ranging characteristics.^36^ As such, the services index that we used may have lacked meaning for our outcome. The unexplained village variance could denote other factors represented within villages, such as school characteristics, which have been found to be more influential on depression among adolescents than neighbourhoods alone.^37^ In either case, the adverse effects found at the village level are likely the result of an unobserved mechanism specific to where these youth live. Other studies have found neighbourhood-level ICCs for depression/depressive symptoms, ranging from 0.4% to 2.9% for adults, and 11% for young children,^38^ although no studies, to our knowledge, report neighbourhood-level variance of depression in Africa.

One potential explanation for regional disparities among boys relates to inequalities in household economic opportunities. Mbeya households owned more livestock and grew more cash crops, providing more income-generating opportunities within their households. While overall labour-hours were similar, Iringa boys spent eight fewer hours on economic activities and six additional hours on domestic chores. In Tanzania, the division of labour is highly gendered, wherein women are responsible for domestic duties on top of any income generating activities.^39^ Although less discussed, patriarchal gender stereotypes can also burden men, as fulfilling the masculine role of provider becomes increasingly difficult, particularly in settings with few opportunities.^40^ On further investigation, 43% of Iringa boys reported low internal LOC (compared with 20% in Mbeya), but not poorer self-esteem. Our two-item self-esteem measurement may lack the sensitivity needed to capture dimensions of self-worth in relation to societal pressures. Failing to live up to typical ‘masculine’ cultural expectations may have led to decreased autonomy and poorer mental health.

We hypothesise that factors related to having a romantic partner increased psychological distress, particularly among girls. Among those in a relationship from our sample, 31% of girls reported ever being pregnant, compared with just 4% of single girls. Qualitative findings from the main evaluation cite pregnancy as a source of major stress for girls.^21^ Pregnancy in adolescence can lead to disrupted schooling, relationship difficulties, poorer health, and decreased economic stability.^41^

While a recent study in Africa explored associations of school enrolment and income-generating activities with depression among adolescents in Tanzania, with results suggesting income-generating activities were associated with depression in fully adjusted models,^9^ they did not explore the additional burden of employment on in-school youth, nor did they examine associations of depression among youth neither in education, employment or training (NEET). Similar to our results, a study conducted in Mexico found higher odds of mental health disorders for employed youth, youth who worked and studied, and NEET youth, compared with those who exclusively studied.^42^ To our knowledge, this is the first study in Tanzania or neighbouring countries to measure how this state of social and economic exclusion associates with poorer mental health during adolescence, only after NEET youth have reached adulthood.

Climate change has been cited as the largest threat to global health in the 21st century.^43^ Despite increased recognition of the negative effects that climate change has on mental health, there are remarkably few studies examining this relationship,^44^ particularly for adolescents.^45^ As populations in Africa are disproportionately at risk for the effects of climate change,^43^ it is critical to include their experiences in the evidence-base. The results here suggest that adolescents affected by extreme precipitation had higher rates of depressive symptoms. Although this result cannot be interpreted as causal, it is an important finding, as severe weather events have been linked to poor mental health outcomes in other populations.^44^

While barriers to care including lack of mental healthcare professionals and services are likely to persist, results from this analysis provide valuable entry points to improve adolescent mental health in low-resource settings. Rather than viewing policies as simple determinants of individual change, community-based interventions can be targeted to areas prone to climate change or economic volatility. Interventions among youth can incorporate components related to sexual and reproductive health, interpersonal relationships, and economic empowerment, and should help youth navigate cultural expectations related to gender norms.

This study has several limitations. First, adolescents in the evaluation lived in households identified as extremely poor using both geographic and village-level targeting. This homogeneity probably led to an underestimation of neighbourhood and economic associations. However, our analysis brings attention to the most at-risk youth within an already vulnerable population, such as the 8% who experienced excessive economic shocks and the 32% affected by extreme precipitation. Second, the use of cross-sectional data does not allow for directional interpretation of results, particularly for time-variant indicators. For example, employment or relationship status may have complex bidirectional relationships with adolescent mental health. However, all determinants were selected based on theory and existing research, and many are persistent and unchanging. By adjusting for a broad range of confounders we believe that these associations are meaningful contributions to the knowledge base. Finally, the lack of temporal associations limited our ability to look at pathways of effects, such as mediating roles of psychosocial indicators. However, as we found strong bivariate associations, we provide context for future research.

In conclusion, our results reinforce that adolescent mental health is associated with diverse, multilevel factors. As social determinants of poor mental health coexist in various domains, effective interventions to improve mental health require an intersectoral approach. These results also highlight the importance of using a gender-focused lens when examining mental health. Future research should better examine how climate change may impact mental health, particularly for African adolescents who represent a large proportion of the at-risk population.

**What is already known on this subject**

- Identifying the social determinants of mental health is crucial to reduce the burden of adolescent depression which profoundly affects youth in real time as well as along the life path.
- While the evidence on social determinants of health in African contexts continues to grow, most research focuses on adults, or on specialised subgroups of adolescents, such as HIV-positive, exclusively in-school, and pregnant/recently pregnant populations.

**What this study adds**

- While our results support that multidimensional factors are associated with poor mental health among Tanzanian adolescents, girls and boys have differing risk and protective factors.
- Our results indicate that extreme precipitation is associated with higher levels of depressive symptoms, with stronger effects for boys, and that being in a romantic relationship may disproportionately affect the mental health of girls due to added stressors. Lack of economic opportunities may also lead to worse mental health, especially among boys who may feel societal pressure to be engaged in income-generating activities.

## Data Availability

No data are available. Data analysed for this study are not publicly available but may become available, subject to government approval, after completion of the main Cash Plus evaluation.
