# Supplementary material for: Exploring multilevel social determinants of depressive symptoms for Tanzanian adolescents: evidence from a cross-sectional study
Source: J Epidemiol Community Health. 2021 Mar 29;75(10):944–54. doi: 10.1136/jech-2020-216200 (PMC8458088; doi:10.1136/jech-2020-216200)
Supplement: Supplementary data [file jech-2020-216200supp001.pdf]

**Supplemental table 1: Centre for Epidemiological Studies Depression Scale 10-item scale items**
All items range from 1 to 4: [1 = rarely (< 1 day), 2 = some or a little of the time (1–2 days); 3 = occasionally or a moderate amount of time (3–4 days); 4 = most or all of the time (5–7 days)]
(1) Did you sleep well? (reverse coded)
(2) Were you happy? (reverse coded)
(3) Did you have trouble concentrating?
(4) Did you feel hopeful about the future? (reverse coded)
(5) Did you feel that everything you did was an effort?
(6) Did you feel lonely?
(7) Did you feel depressed?
(8) Did you feel that you could not get going?
(9) Were you bothered by things that don't usually bother you?
(10) Did you feel fearful?

Supplemental table 2: Definitions and variable construction of potential social determinants, by domain

**Domain**<br>Lund et al. (2018) definition	**Determinant**	**Variable type**	**Survey source**	**Definition**
**Demographic**<br>The specific demographic characteristics of populations that convey risk for, or protection from, mental illness	Sex	Categorical:<br>1=Male<br>2=Female	Youth	Sex as reported by youth
	Age in years	Integer	Youth	Age as reported by youth
	Female-headed household	Binary	Household	Sex of the household head as reported by the household respondent
	Five or more household members	Binary	Household	Dichotomized household size based on household roster. The cutoff (5 or more vs. less than 5) was selected based on the mean number of household members (4.96).
	Region	Categorical:<br>Iringa=1<br>Mbeya=2	Village	Preloaded categories of the study districts were recategorized by their region. Mufindi/Mafinga districts are part of Iringa region and Rungwe/Busokelo districts Mbeya.
**Economic**<br>Factors relating to the production, consumption, and transfer of wealth that convey risk for, or protection from, mental illness	**Indicator**	**Type**	**Survey**	**Definition**
	Wealth level of household	Categorical:<br>Richest=1<br>Middle=2<br>Poorest=3	Household	The wealth index was obtained with factor analysis, considering dwelling characteristics, livestock, and durable ownership. We created levels of wealth based on tertiles of all 1,946 households in the interviewed sample.
	Number of economic shocks	Count<br>(zero to 4; 5 or more)	Household	Sum of negative economic shocks experienced by the household during the 12 months prior to baseline (high levels of crop/livestock disease; low prices for agricultural output; high prices for agricultural inputs; unusually high cost of food; loss of outside aid/remittances; reduced earnings from non-agricultural household business; failure of non-agricultural household business; reduced income of salaried household members; lost employment by salaried household members; serious illness/injury of household members; birth in the household; death of income earner; death of other household members; theft of money/valuables/assets/agricultural output). Five or more shocks were grouped due to distribution.
	Youth owns a cellphone	Binary	Youth	Cellphone ownership as reported by youth
**Neighbourhood**<br>Characteristics of a community that convey risk for, or protection from, mental illness, over and above what is attributable to the individual characteristics of community members	**Indicator**	**Type**	**Survey**	**Definition**
	Access to services level	Categorical:<br>Lowest=1<br>Middle=2<br>Highest=3	Village	The access to services index was obtained with factor analysis, considering access to basic services within the village, such as schools, health services, clean water sources, electricity, paved roads, markets, and NGOs. We created levels of access based on tertiles of all 130 villages in the sample.
**Environmental**<br>Serious disruptions of the functioning of a community that exceed its ability to cope by use of its own resources and convey risk for mental illness	**Indicator**	**Type**	**Survey**	**Definition**
	Household experienced drought and/or flood	Binary	Household	Whether household respondent reported any negative shocks caused by drought/irregular rains or flood/landslides during the past 12 months.
	Household experienced livestock or crop disease (including pests)	Binary	Household	Whether household respondent reported any negative shocks caused by high levels of livestock disease or high levels of crop pests/crop disease during past 12 months
**Social and cultural**: ways in which the organisation of society, social interactions, and relationships affect risk of, and protection from, mental illness	**Indicator**	**Type**	**Survey**	**Definition/**
	Education/ employment status	Categorical:<br>1=Attending school/training<br>2=Engaged in paid work	Youth	Based on youth response for school or training attendance at time of survey and whether youth was engaged in any paid work outside of the household. Paid work excludes any agricultural, livestock, or non-farm business activities within the household duties.

Education, social cohesion, social capital, culture, and social class		3=School & paid work<br>4=Neither		
	Has a partner	Binary	Youth	Includes all married youth (religious/nonreligious/polygamous/monogamous of marriage), youth cohabitating with a partner (monogamous or polygamous), and youth who reported having a girlfriend or boyfriend.
	Weekly religious attendance	Binary	Youth	Indicator based on how often youth attends church, mosque, other religious ceremonies: weekly vs. almost every week, about once a month, only special occasions, or never<br><br>Source: “Gallup world poll” https://media.gallup.com/dataviz/www/wp_questions_white.pdf
	Adverse childhood experiences	Scale<br>(0-1, 2, 3, 4, or 5 or more)	Youth	Scale ranges from 0-8 (based on categories below). Five or more were grouped due to distribution. Questions which asked for frequency were coded 0 for never/rarely and 1 for any other frequency.<br><br>Experienced emotional abuse<br>Experienced physical neglect<br>Parent/guardian/caregiver divorced or deceased<br>Witnessed domestic violence in the household<br>Alcohol and/or drug abuser lived in the household<br>Mentally ill household member lived in the household<br>Experienced bullying<br>Experienced community violence<br><br>Source: subset of questions selected from “Adverse Childhood Experiences International Questionnaire (ACE-IQ)” https://www.who.int/violence_injury_prevention/violence/activities/adverse_childhood_experiences/en/
	Social support levels	Tertiles<br>Low=1<br>Moderate=2<br>High=3	Youth	Based on averaged score (1-5) across four questions of familial or friend support where higher scores indicate higher social support. Tertiles were created from average using the sample population.<br><br>Answers range from one ‘Strongly disagree’ to five ‘Strongly agree’<br>*I have friends with whom I can share my joys and sorrows*<br>*I can talk about my problems with my friends*<br>*I get the help and support I need from my family*<br>*My family is willing to help me make decisions*<br><br>Source: Modified version of MSPSS (Zimmet 1990)

Supplemental table 3: Definitions and variable construction of psychosocial indicators

Indicator	Variable type	Survey source	Definition
Self-esteem level	Tertiles<br>Low=1<br>Moderate=2<br>High=3	Youth	Based on averaged score (1-5) across two items from Rosenberg's self-esteem scale questions where higher scores indicate higher self-esteem. Tertiles were created from average using the sample population.<br><br>Answers range from one 'Strongly disagree' to five 'Strongly agree'<br>*You feel that you have many good qualities*<br>*On the whole, you are satisfied with yourself.*<br><br>Source: Modified version of MSPSS (Zimmet 1990)
Poor self-perceived quality of life (QOL)	Binary	Youth	Youth were considered having a "poor" QOL with a score lower than the regional mean on a ten-point scale survey question:<br><br>*Imagine a ladder where on the bottom, the first step, represents the worst possible life for you and the highest step, the tenth, represents the best possible life for you. On which step of the ladder would you say you are today?*
Locus of control (LOC) level	Tertiles<br>Low=1<br>Moderate=2<br>High=3	Youth	Based on averaged score (1-5) across 5-items from Levenson's multidimensional LOC scale20. A higher locus of control indicates more internal control. Tertiles were created from average using the sample population.<br>Answers range from one 'Strongly disagree' to five 'Strongly agree'<br>*It's not always wise for you to plan too far ahead because many things turn out to be a matter of good or bad fortune (reverse coded)*<br>*Your life is determined by your own actions*<br>*When you get what you want, it is usually the result of your own actions*<br>*You feel like what happens in your life is mostly determined by others (reverse coded)*<br>*Getting what you want requires pleasing the influential people (reverse coded)*

Supplemental table 4: Associations of potential determinants and depressive symptoms on the full sample, all multivariate models

	Model 1		Model 2		Model 3		Model 4	
**Variable**	**Estimate (95% CI)**	**p-value**	**Estimate (95% CI)**	**p-value**	**Estimate (95% CI)**	**p-value**	**Estimate (95% CI)**	**p-value**
**Male (ref=female)**	0.12 (-0.26 to 0.50)	0.543	0.06 (-0.32 to 0.44)	0.753	0.70 (0.30 to 1.09)	0.001	0.70 (0.31 to 1.10)	<0.001
**Age in years (ref=14)**								
15	0.60 (0.01 to 1.20)	0.045	0.62 (0.03 to 1.22)	0.040	0.58 (-0.00 to 1.16)	0.050	0.60 (0.03 to 1.18)	0.039
16	0.98 (0.38 to 1.59)	0.001	0.94 (0.33 to 1.54)	0.003	0.66 (0.06 to 1.26)	0.030	0.73 (0.14 to 1.32)	0.016
17	1.40 (0.78 to 2.02)	<0.001	1.41 (0.81 to 2.02)	<0.001	0.83 (0.20 to 1.45)	0.009	0.95 (0.33 to 1.58)	0.003
18	1.56 (0.81 to 2.31)	<0.001	1.55 (0.87 to 2.24)	<0.001	0.60 (-0.13 to 1.34)	0.106	0.86 (0.10 to 1.62)	0.027
19	1.92 (1.11 to 2.73)	<0.001	1.88 (1.14 to 2.62)	<0.001	0.78 (-0.02 to 1.58)	0.055	1.09 (0.26 to 1.92)	0.010
**Large household**	0.31 (-0.09 to 0.70)	0.130	0.40 (-0.00 to 0.80)	0.050	0.47 (0.09 to 0.86)	0.016	0.37 (-0.01 to 0.76)	0.057
**Female-headed**	0.08 (-0.33 to 0.50)	0.696	0.14 (-0.27 to 0.56)	0.505	0.15 (-0.25 to 0.56)	0.459	0.16 (-0.24 to 0.56)	0.426
**Wealth level (ref=richest)**								
Middle	0.50 (0.01 to 0.99)	0.046					0.45 (-0.02 to 0.93)	0.063
Poorest	0.60 (0.06 to 1.13)	0.028					0.47 (-0.05 to 0.99)	0.076
**Economic shocks (ref=0)**								
1	0.07 (-0.46 to 0.60)	0.790					0.01 (-0.51 to 0.54)	0.964
2	-0.24 (-0.82 to 0.33)	0.403					-0.39 (-1.03 to 0.24)	0.223
3	-0.14 (-0.81 to 0.53)	0.686					-0.08 (-0.87 to 0.70)	0.834
4	0.12 (-0.71 to 0.95)	0.777					0.35 (-0.58 to 1.28)	0.463
5+	2.68 (1.91 to 3.46)	<0.001					2.40 (1.48 to 3.32)	<0.001
**Youth owns cell phone**	-0.06 (-0.63 to 0.50)	0.824					-0.44 (-1.01 to 0.12)	0.123
**Services level (ref=highest)**								
Middle			-0.00 (-0.72 to 0.71)	0.994			-0.03 (-0.72 to 0.65)	0.927
Low			0.10 (-0.63 to 0.83)	0.787			0.02 (-0.68 to 0.72)	0.947
**Drought/flood**			0.91 (0.49 to 1.33)	<0.001			0.76 (0.36 to 1.17)	<0.001
**Livestock/crop disease**			0.14 (-0.26 to 0.54)	0.490			-0.25 (-0.78 to 0.28)	0.354
**Education/employment status (ref=attending school/training)**								
Paid work					0.56 (-0.10 to 1.23)	0.098	0.56 (-0.10 to 1.23)	0.098
School and paid work					0.95 (-0.09 to 1.98)	0.073	1.07 (0.05 to 2.09)	0.040
Neither					0.71 (0.22 to 1.19)	0.004	0.73 (0.24 to 1.22)	0.003
**Has a partner**					1.77 (1.25 to 2.29)	<0.001	1.82 (1.30 to 2.33)	<0.001
**Social support (ref=high)**								
Moderate					1.27 (0.81 to 1.73)	<0.001	1.26 (0.80 to 1.71)	<0.001
Low					2.36 (1.89 to 2.83)	<0.001	2.27 (1.81 to 2.74)	<0.001

**Village variance (SE)**	1.51 (0.345)	1.56 (0.354)	1.66 (0.359)	1.41 (0.325)
**Intraclass correlation coefficient%**	6.5%	6.5%	7.4%	6.5%

Multivariate regressions tested associations between domains and depressive symptoms (demographic domain was included in all models; neighborhood and environmental domains were combined; final model includes all domains). Models adjust for stratification variables and employ multilevel methods (linear mixed models) to adjust for clustering of individual depressive symptoms within and between communities. Indicators from wave 2 (adverse childhood experiences and religious attendance) were excluded from multivariate models.

Supplemental table 5: Associations of potential determinants and depressive symptoms among females, all multivariate models

	**Model 1**		**Model 2**		**Model 3**		**Model 4**	
**Variable**	**Estimate (95% CI)**	**p-value**	**Estimate (95% CI)**	**p-value**	**Estimate (95% CI)**	**p-value**	**Estimate (95% CI)**	**p-value**
**Age in years (ref=14)**								
15	0.17 (-0.65 to 0.99)	0.684	0.18 (-0.65 to 1.02)	0.666	0.28 (-0.52 to 1.08)	0.494	0.34 (-0.45 to 1.13)	0.401
16	0.75 (-0.12 to 1.62)	0.092	0.61 (-0.28 to 1.50)	0.176	0.40 (-0.46 to 1.25)	0.365	0.57 (-0.27 to 1.41)	0.184
17	0.97 (0.06 to 1.87)	0.036	1.15 (0.25 to 2.04)	0.012	0.49 (-0.42 to 1.40)	0.295	0.50 (-0.41 to 1.41)	0.278
18	1.70 (0.59 to 2.82)	0.003	2.07 (1.03 to 3.11)	<0.001	0.92 (-0.18 to 2.02)	0.103	0.98 (-0.15 to 2.11)	0.089
19	1.42 (0.22 to 2.61)	0.020	1.82 (0.73 to 2.91)	0.001	0.28 (-0.91 to 1.47)	0.648	0.39 (-0.85 to 1.62)	0.539
**Large household**	0.64 (0.06 to 1.22)	0.031	0.71 (0.12 to 1.30)	0.018	0.67 (0.11 to 1.24)	0.020	0.62 (0.06 to 1.18)	0.029
**Female-headed**	0.40 (-0.21 to 1.00)	0.196	0.46 (-0.15 to 1.08)	0.140	0.31 (-0.28 to 0.90)	0.302	0.30 (-0.28 to 0.89)	0.305
**Wealth level (ref=Richest)**								
Middle	0.62 (-0.08 to 1.31)	0.081					0.44 (-0.23 to 1.11)	0.193
Poorest	0.50 (-0.25 to 1.25)	0.194					0.27 (-0.46 to 1.01)	0.464
**Economic shocks (ref=0)**								
1	-0.16 (-0.93 to 0.61)	0.685					-0.36 (-1.13 to 0.40)	0.353
2	-1.10 (-1.94 to -0.26)	0.010					-1.39 (-2.30 to -0.47)	0.003
3	-0.35 (-1.34 to 0.64)	0.486					-0.46 (-1.61 to 0.69)	0.430
4	-1.45 (-2.68 to -0.22)	0.021					-1.24 (-2.62 to 0.14)	0.079
5+	2.57 (1.45 to 3.69)	<0.001					1.96 (0.63 to 3.29)	0.004
**Youth owns cell phone**	0.71 (-0.17 to 1.60)	0.116					0.16 (-0.72 to 1.05)	0.716
**Services level (ref=highest)**								
Middle			0.47 (-0.36 to 1.31)	0.262			0.24 (-0.54 to 1.03)	0.543
Low			0.01 (-0.84 to 0.87)	0.977			-0.18 (-1.00 to 0.63)	0.659
**Drought/flood**			0.59 (-0.05 to 1.22)	0.071			0.42 (-0.19 to 1.02)	0.180
**Livestock/crop disease**			-0.08 (-0.68 to 0.52)	0.798			-0.12 (-0.89 to 0.66)	0.762
**Education/employment status (ref=attending school/training)**								
Paid work					0.34 (-0.93 to 1.61)	0.596	0.26 (-1.00 to 1.53)	0.683
School and paid work					0.02 (-1.80 to 1.84)	0.983	0.24 (-1.55 to 2.04)	0.791
Neither					0.65 (-0.08 to 1.38)	0.079	0.51 (-0.23 to 1.25)	0.178
**Has a partner**					2.34 (1.68 to 3.00)	<0.001	2.23 (1.58 to 2.88)	<0.001

**Social support (ref=high)**								
Moderate					0.84 (0.14 to 1.55)	0.019	0.86 (0.17 to 1.56)	0.015
Low					2.23 (1.55 to 2.92)	<0.001	2.20 (1.52 to 2.88)	<0.001
**Village variance (SE)**	1.12 (0.468)		1.16 (0.484)		1.21 (0.465)		1.02 (0.438)	
**Intraclass correlation coefficient%**	4.9%		4.9%		5.6%		4.9%	

Multivariate regressions tested associations between domains and depressive symptoms (demographic domain was included in all models; neighborhood and environmental domains were combined; final model includes all domains). Models adjust for stratification variables and employ multilevel methods (linear mixed models) to adjust for clustering of individual depressive symptoms within and between communities. Indicators from wave 2 (adverse childhood experiences and religious attendance) were excluded from multivariate models.

Supplemental table 6: Associations of potential determinants and depressive symptoms among males, all multivariate models

	Model 1		Model 2		Model 3		Model 4	
**Variable**	**Estimate (95% CI)**	**p-value**	**Estimate (95% CI)**	**p-value**	**Estimate (95% CI)**	**p-value**	**Estimate (95% CI)**	**p-value**
**Age in years (ref=14)**								
15	0.92 (0.10 to 1.73)	0.027	0.95 (0.13 to 1.76)	0.023	0.81 (0.01 to 1.62)	0.049	0.85 (0.05 to 1.64)	0.037
16	1.12 (0.31 to 1.92)	0.006	1.12 (0.32 to 1.93)	0.006	0.85 (0.05 to 1.66)	0.038	0.93 (0.14 to 1.73)	0.022
17	1.42 (0.61 to 2.24)	0.001	1.39 (0.60 to 2.18)	0.001	0.95 (0.13 to 1.78)	0.024	1.21 (0.38 to 2.04)	0.004
18	1.27 (0.30 to 2.24)	0.011	1.11 (0.23 to 2.00)	0.013	0.44 (-0.51 to 1.39)	0.368	0.75 (-0.24 to 1.74)	0.138
19	2.20 (1.13 to 3.27)	<0.001	1.98 (1.00 to 2.96)	<0.001	1.32 (0.27 to 2.37)	0.014	1.72 (0.62 to 2.81)	0.002
**Large household**	-0.26 (-0.77 to 0.26)	0.332	-0.12 (-0.63 to 0.40)	0.656	0.02 (-0.49 to 0.53)	0.944	-0.10 (-0.60 to 0.41)	0.703
**Female-headed**	-0.23 (-0.77 to 0.31)	0.401	-0.17 (-0.71 to 0.37)	0.532	-0.08 (-0.61 to 0.45)	0.761	-0.02 (-0.55 to 0.51)	0.933
**Wealth level (ref=Richest)**								
Middle	0.31 (-0.36 to 0.97)	0.368					0.39 (-0.26 to 1.04)	0.239
Poorest	0.47 (-0.25 to 1.19)	0.199					0.42 (-0.29 to 1.12)	0.245
**Economic shocks (ref=0)**								
1	0.25 (-0.45 to 0.95)	0.479					0.45 (-0.25 to 1.15)	0.209
2	0.41 (-0.33 to 1.16)	0.276					0.57 (-0.27 to 1.41)	0.181
3	0.09 (-0.79 to 0.97)	0.838					0.59 (-0.46 to 1.63)	0.271
4	1.52 (0.45 to 2.59)	0.005					1.89 (0.69 to 3.09)	0.002
5+	2.21 (1.17 to 3.25)	<0.001					2.43 (1.19 to 3.67)	<0.001
**Youth owns cell phone**	0.31 (-0.36 to 0.97)	0.368					-0.61 (-1.32 to 0.10)	0.094
**Services level (ref=highest)**								
Middle			-0.49 (-1.54 to 0.57)	0.366			-0.32 (-1.34 to 0.70)	0.533
Low			0.18 (-0.89 to 1.25)	0.739			0.22 (-0.82 to 1.26)	0.683
**Drought/flood**			1.15 (0.61 to 1.69)	<0.001			1.03 (0.51 to 1.56)	<0.001
**Livestock/crop disease**			0.14 (-0.37 to 0.65)	0.587			-0.60 (-1.30 to 0.09)	0.088

**Education/employment status (ref=attending school/training)**						
Paid work			0.68 (-0.09 to 1.46)	0.084	0.63 (-0.15 to 1.40)	0.113
School and paid work			1.37 (0.16 to 2.58)	0.027	1.41 (0.22 to 2.61)	0.021
Neither			0.58 (-0.05 to 1.22)	0.073	0.66 (0.02 to 1.31)	0.043
**Has a partner**			0.91 (0.07 to 1.75)	0.034	1.07 (0.23 to 1.90)	0.012
**Social support (ref=high)**						
Moderate			1.34 (0.76 to 1.92)	<0.001	1.31 (0.74 to 1.88)	<0.001
Low			2.10 (1.46 to 2.74)	<0.001	2.11 (1.47 to 2.74)	<0.001
**Village variance (SE)**	3.88 (0.798)	3.91 (0.781)	4.13 (0.804)		3.63 (0.744)	
**Intraclass correlation coefficient%**	16.8%	16.9%	18.3%		16.8%	

Multivariate regressions tested associations between domains and depressive symptoms (demographic domain was included in all models; neighborhood and environmental domains were combined; final model includes all domains). Models adjust for stratification variables and employ multilevel methods (linear mixed models) to adjust for clustering of individual depressive symptoms within and between communities. Indicators from wave 2 (adverse childhood experiences and religious attendance) were excluded from multivariate models.
